# Linking event-related potentials and structural MRI in the aging brain

**DOI:** 10.3389/fnagi.2026.1945797

**Published:** 2026-10-19

**Authors:** Yajya Rishi, Raj Attariwala, Ryan C. N. D’Arcy

**Affiliations:** 1 Faculty of Applied Sciences, Simon Fraser University, Burnaby, BC, Canada; 2 AIM Medical Imaging, Vancouver, BC, Canada; 3 Voxelwise Imaging Technology, Vancouver, BC, Canada; 4 Centre for Neurology Studies, HealthTech Connex, Vancouver, BC, Canada; 5 DM Centre for Brain Health, University of British Columbia, Vancouver, BC, Canada

**Keywords:** brain aging, EEG, event-related potentials, healthy aging, multimodal neuroimaging, P300, structural MRI, structure–function coupling

## Abstract

Aging alters both brain structure and electrophysiology, which can be measured non-invasively by structural magnetic resonance imaging (MRI) and event-related potentials (ERPs). On average, gray matter declines steadily from early adulthood, whereas white matter increases into midlife before declining. With respect to ERPs, the well-established P300 response shows reduced amplitude and delayed latency from early adulthood onward. Few studies test whether structural MRI and ERP measures correlate within the same individuals, and those that do disagree over which tissue tracks which ERP change. While ERP changes are not coupled to structural changes in a fixed manner, atrophy in gray and white matter tissue correlate with ERP response decay, and a clear pattern of structure–function coupling emerges with age. This dynamic in turn is magnified by age and disease. Technological advances are now enabling structural MRI and portable ERP to be collected from the same individual in a single visit, with machine learning able to model the two modalities jointly rather than separately. While clinical cohorts have historically been the starting point, healthy normative data sampled across the adult age range represents the foundational baseline. Establishing this coupling directly is the critical first step toward integrating MRI and ERP as mutually informative rather than parallel measures of the aging brain. Accordingly, this brief review assembles the evidence as a conceptual framework for linking MRI and ERP changes in healthy aging to support preclinical detection of cognitive decline, now enabled through recent technological advances.

## 1 Introduction

Aging brings gradual changes to the brain that may contribute to declines in cognitive functions such as memory, attention, and executive function, across the adult lifespan (MacDonald and Pike, 2021; van Dinteren et al., 2014). These changes appear in two complementary measures of brain aging, structural neuroimaging and electrophysiology. Magnetic resonance imaging (MRI) characterizes brain structure, while event-related potentials (ERPs) capture neural activity.

MRI shows age-related reductions in both cortical thickness and gray matter (GM) and white matter (WM) volume, most pronounced in frontal association cortex that supports higher-order cognition (Fjell and Walhovd, 2010; MacDonald and Pike, 2021). ERP measures show a corresponding functional decline, slowed neural processing and weaker stimulus evaluation, captured by responses such as the P300, an index of basic attention, and the N400, an index of cognitive processing, among others (Friedman, 2003; van Dinteren et al., 2014).

In aging research, these two measures have largely been studied separately, in part because few datasets combine both MRI and ERP obtained from the same individuals. The studies that correlate the two directly are few and draw mostly from clinical cohorts, leaving the relationship in healthy aging poorly characterized. This relationship is most informative in healthy aging, where structural and functional changes are already underway before clinical symptoms appear (Risacher and Saykin, 2013; Paitel et al., 2021).

Structural MRI is more often paired with functional MRI, but the blood-oxygen-level-dependent (BOLD) signal is hemodynamic. Aging alters baseline cerebral blood flow, cerebrovascular reactivity and neurovascular coupling (D'Esposito et al., 2003), hence age differences in BOLD are not necessarily neural, and when task-related BOLD is scaled by an index of vascular reactivity much of the apparent age effect does not survive (Tsvetanov et al., 2015; Henson et al., 2024). A comparable confound applies to ERPs. Age-related volume loss can reduce ERP amplitude and alter its scalp distribution through two routes, a neural one in which less tissue generates the response, and a geometric one in which the cortex sits further from the electrodes and expanded CSF shunts current away. Modeling this shrinkage shows the geometric route produces only a small share of the attenuation at the limit of plausible atrophy, against the much larger reductions reported in healthy older adults, where neural change contributes alongside it (He et al., 2021). Given the limits of each modality, combining MRI and EEG appears better suited to measuring age-related structural and functional changes than either measure alone.

Acquiring structural MRI and ERP together is now practical at scale. This is an important advance in terms of practical applications for early screening of age-related cognitive impairment. Given that MRI (and related techniques like fMRI) are not portable, ERP’s can potentially provide a valuable screening tool if a relationship between the two imaging modalities can be empirically validated. Compact, point-of-care ERP acquisition, exemplified by standardized frameworks such as Brain Vital Signs, can be paired with rapid structural MRI to yield a matched multimodal dataset from the same individual (Ghosh Hajra et al., 2016, 2018). Machine-learning methods can then integrate structure and function across the whole brain, recovering the distributed coupling that earlier region-by-region analyses were underpowered to resolve.

Accordingly, this brief review synthesizes the small but growing literature correlating MRI and ERP directly. It asks whether the variability across these findings reflects a single structure–function relationship that shifts systematically with age, or whether the findings represent independent results without common cause. This review argues for the former, organizing the scattered findings into a conceptual framework that specifies what a normative model of healthy aging would require, namely the baseline against which individual structure and function can be read together for preclinical detection of cognitive decline.

## 2 Structural brain aging

GM volume declines about 0.83 percent per year in healthy older adults (Crivello et al., 2014). WM behaves differently, largely preserved through midlife before declining rapidly in the final decades (Allen et al., 2005; Ge et al., 2002). Whole-brain decline can be summarized as a single burden score, such as the Brain Atlas and Lesion Index, which combines atrophy and lesion load into a measure that rises with age and disease severity (Zhang et al., 2011; Guo et al., 2017). Such indices, however, average over the distinct trajectories of the two tissue classes. GM volume declines approximately linearly from young adulthood, with no midlife peak (Ge et al., 2002; Walhovd et al., 2011; MacDonald and Pike, 2021). WM volume instead follows a quadratic trajectory, rising through continued myelination into the fourth or fifth decade before peaking and entering a decline that steepens with age (Ge et al., 2002; Walhovd et al., 2011; MacDonald and Pike, 2021). The divergent aging trajectories of GM and WM appear to originate in development. Current evidence indicates that GM growth is complete before early adulthood (Ge et al., 2002; Walhovd et al., 2011), whereas WM continues to mature into the third decade (Bethlehem et al., 2022). Across adulthood the trajectories suggest GM is in decline through synaptic and dendritic loss (Morrison and Hof, 1997), while WM still accrues myelin through the protracted myelination of association pathways before breakdown begins (Bartzokis, 2004). Both tissues remain subject to experience-dependent plasticity (Zatorre et al., 2012; Frizzell et al., 2020) and to vascular change, to which WM is particularly susceptible (Pantoni, 2010). Diffusion-based measures capture a different WM property, its microstructural integrity, which changes earlier than volume, with fractional anisotropy (FA) peaking in the late twenties (Westlye et al., 2010). Across measures, mid-life emerges as an inflection point where several regional trajectories shift toward accelerated decline.

Atrophy varies regionally, with association cortex declining earliest and most steeply. WM declines across tracts rather than concentrating in regions, with the steepest loss in prefrontal pathways (Resnick et al., 2003; Raz et al., 2005; Westlye et al., 2010; MacDonald and Pike, 2021). Frontal cortex, and lateral prefrontal regions in particular, show the steepest GM loss, identified consistently across study designs and by both volume and cortical thickness, with decline extending to the inferior parietal, cingulate, and insular cortices (Salat et al., 2004; Raz et al., 2005; Good et al., 2001; Resnick et al., 2003; Fjell and Walhovd, 2010; Lemaitre et al., 2012; MacDonald and Pike, 2021). The hippocampus is among the most affected subcortical regions in later life, with decline accelerating from the mid-fifties onward, accentuated by vascular risk factors (Raz et al., 2005; Fjell et al., 2013). Other subcortical structures such as the putamen and nucleus accumbens decline more linearly (Dima et al., 2022; Fjell and Walhovd, 2010; MacDonald and Pike, 2021). At the relatively preserved end are the parahippocampal cortex, primary sensory cortices, and the amygdala (Good et al., 2001; Salat et al., 2004).

Reported sex differences are modest. Larger absolute brain volume in males reflects intracranial volume and largely disappears once volumes are normalized to it (Ge et al., 2002; Good et al., 2001), while reports of steeper GM decline in men remain inconsistent in both magnitude and direction (Ravndal et al., 2025; Raz et al., 2005).

## 3 Electrophysiological brain aging

The P300 and N400 have been the primary focus of ERP research in cognitive aging, the former across attention and oddball paradigms, the latter in language and semantic processing, while earlier and smaller components such as the P50, N100, P200, and MMN have been reported more sparsely (Polich, 1996; Kok, 2000; Friedman, 2003). Across this literature, age-related changes have been described along three measurable properties of the ERP, namely latency, amplitude, and scalp topography, with patterns that differ by component.

Latency changes are the most consistently reported, seen as a graded slowing with age. Normative work found that age-related slowing scaled with each component’s baseline latency, least for the N100 and greatest for the P300 (Goodin et al., 1978), consistent with generalized neural slowing in aging. P300 latency increases approximately linearly at 1–2 ms/year (Pfefferbaum et al., 1984; Polich, 1996), with larger studies showing the rate of slowing accelerating after the sixth decade (Anderer et al., 1996; van Dinteren et al., 2014). N400 latency shows a similar prolongation in semantic tasks, increasing at just under 2 ms/year (Kutas and Iragui, 1998; Ghosh Hajra et al., 2016), while N100 latency has remained comparatively preserved across the adult lifespan (Goodin et al., 1978; Anderer et al., 1996).

Amplitude patterns are more heterogeneous across components, varying in magnitude and sometimes in direction. Amplitude reductions with age are most consistently reported for the P300, and to a lesser extent the N400 (Anderer et al., 1996; Kutas and Iragui, 1998; van Dinteren et al., 2014; Ighalo et al., 2024). Findings for the early N100 and P200 components are less consistent, with some studies reporting a modest frontal increase with age (Anderer et al., 1996; Goodin et al., 1978). Differences across studies in reference scheme, filtering, and amplitude measurement further contribute to this variability.

Topographic redistribution with age is most extensively documented for the P300. In younger adults the P300 is maximal over parietal cortex; with age this distribution flattens and becomes progressively equipotential, with frontal contributions becoming proportionally more prominent (Pfefferbaum et al., 1984; Anderer et al., 1996; Friedman, 2003). This redistribution has been attributed in part to a reduction in an overlapping frontal-negative slow wave, which may account for much of the apparent shift rather than a true change in the P300 (Pfefferbaum et al., 1984). It has also been attributed to increased frontal engagement in older adults, sometimes interpreted as compensatory recruitment (Friedman, 2003). Not all components redistribute, however. The N100 retains a comparatively stable distribution, anchored over central sites across age (Anderer et al., 1996). The N400 similarly holds its posterior, slightly right-lateralized distribution across decades, even as its amplitude weakens and its latency lengthens (Kutas and Iragui, 1998; Federmeier et al., 2003).

Read as a sequence, the N100, P300 and N400 index sensory, attentional and semantic processing (Ghosh Hajra et al., 2016). Across the studies reviewed here, aging appears to affect later attentional and integrative operations more than early sensory encoding. ERP reductions do not, however, map onto single cognitive deficits, since a smaller P300 may reflect diminished attentional resource, reorganized processing, or recording geometry, and these are not mutually exclusive. In particular the P300 and N400 are further affected in cognitive impairment, where delayed latencies and reduced amplitudes are more pronounced in mild cognitive impairment and Alzheimer’s disease (Joyal et al., 2020; Paitel et al., 2021), with N400 latency in care home residents roughly 130 ms longer in those most severely impaired on the Mini-Mental State Examination than in those least impaired (Ighalo et al., 2024).

## 4 Linking structure and function

A small but expanding literature has examined how scalp-recorded ERPs and structural MRI co-vary with age. The evidence is drawn largely from two distinct cohort types, healthy aging samples and clinical cohorts. Summarized in Table 1, the P300 dominates as the principal, and frequently the sole, ERP measure, while earlier sensory components such as the N100, and the semantic N400, are rarely examined. Across these studies, several patterns recur.

**Table 1:** Studies directly linking ERP measures to structural MRI in healthy adult aging and aging-related clinical cohorts.

Authors (Year)	Participants	ERP task	ERP component	MRI metric	Main finding(s)
Fjell et al. (2007)	Healthy lifespan, *n* = 72 (35 young, *M* = 33.7 yr.; 37 elderly, *M* = 69.5 yr); range 20–88 yr.	Visual three-stimulus oddball	P3a, P3b (amplitude + latency)	Whole-brain vertex-wise cortical thickness; hippocampal and global WM volumes	Elderly only: larger P3a amplitude with thicker left temporoparietal/parietal cortex; shorter P3b latency with thicker frontal/orbitofrontal cortex. Near-zero effects in young.
Pergher et al. (2019)	Healthy older adults; ERP *n* = 36 (young-old 52–64 yr., *n* = 18; old 65–76 yr., *n* = 18); MRI subgroup *n* = 16 (old only)	Visual N-back (1-, 2-, 3-back)	P300 (amplitude + latency)	Whole-brain VBM, GM	Older > young-old: smaller P300 amplitude, longer latency. *n* = 16 MRI subgroup: positive correlations between P300 amplitude/latency and GM in left post-central gyrus, left lingual gyrus, middle temporal gyrus, left supramarginal gyrus, thalamus.
Riedel et al. (2024)	Healthy older adults, *n* = 56 (mean 67.1 ± 4.9 yr)	Visual letter-matching (Dimond)	N170 latency; interhemispheric transfer time (IHTT)	ROI corpus callosum (genu, body, splenium); DTI fractional anisotropy	Slower IHTT with lower FA in splenium (*r* = −0.29). Genu, body ns. ~8% of variance.
Cardenas et al. (2005)	Healthy adults, midlife, *n* = 59 (mean 42 ± 8 yr.; 18 females, 41 male)	Visual three-stimulus oddball	P3a, P3b (latency only; Cz)	Whole-brain GM/WM/CSF segmentation + subcortical ROIs (caudate, lenticular, thalamus, hippocampus); voxel-wise deformation morphometry	Longer P3b latency with smaller WM, thalamus, lenticular nucleus; larger CSF. Voxel-wise contractions in thalamus, frontal WM, corpus callosum. No GM relationship to P3b latency.
Ford et al. (1994)	Healthy adult men only, *n* = 28; ages 21–60 yr.	Three auditory paradigms (automatic, effortful, combined)	P300 (amplitude + latency)	Lobar ROIs (frontal, parietal, temporal GM/WM/CSF) via geometric quadrants; posterior STG	Larger P300 amplitude with greater frontal GM (automatic task), parietal GM (effortful task), or both (combined). GM only (not WM/CSF). Effects attenuated when age partialed out.
Hoppstädter et al. (2013)	Healthy older adults + amnestic MCI; *n* = 14 aMCI (mean 67.8 yr) + *n* = 10 controls (mean 68.0 yr)	Verbal yes/no recognition memory	Frontal and parietal old/new effects (amplitude only)	Whole-brain VBM, GM	aMCI: bilateral medial temporal GM loss. Across-group: larger frontal old/new amplitude with greater left medial temporal cortex and right fusiform GM. Recollection effect absent in both groups.
Braverman et al. (2015)	Mixed clinical aging cohort (memory loss, trauma, headaches; smaller AD, PD, stroke, alcoholism, ADHD subgroups), *n* = 169; age 19–90 yr. (mean 56.3 ± 13.5 yr)	Auditory oddball	P300 (amplitude + latency)	Automated ROI atrophy (binary yes/no): hippocampus, lateral and inferior lateral ventricles; qualitative ratings (WM ischemia, demyelination, frontal-lobe FA)	Longer P300 latency with temporal atrophy (marginal). Higher P300 amplitude with less bilateral atrophy and less demyelination. Weak fits (AUC 0.62–0.76; R^2^ ≈ 0.04–0.14).
Fusar-Poli et al. (2011)	Clinical, prodromal psychosis; *n* = 39 ARMS (mean 24.5 yr) + *n* = 41 controls (mean 25.9 yr); ERP subsample *n* = 28 ARMS + *n* = 13 controls	Auditory oddball	P300 amplitude only (Pz)	Whole-brain VBM, WM only	ARMS: reduced P300 amplitude. Whole sample: larger P300 amplitude with greater genu of corpus callosum WM (*R* = 0.35). Within ARMS: larger P300 with greater right supramarginal/arcuate fasciculus WM. Right MFG group × WM × P300 interaction: positive in controls, negative in ARMS (smaller P300 with greater WM).
Egan et al. (1994)	Clinical, chronic schizophrenia; *n* = 16 medicated patients (mean 30.3 yr) + matched controls	Auditory and visual oddball	N100, N200, P300 (amplitude + latency)	ROI area/volume (single-slice): hippocampus, amygdala, third and lateral ventricles, temporal and prefrontal lobes	Within patients: larger visual P300 amplitude with greater right hippocampal area. N200 amplitude correlated with left hippocampal area. Smaller auditory P300 with greater parietal area and ventricular volume; smaller visual P300 with greater prefrontal volume (Interpreted as volume conduction.)

Given the scarcity of healthy-aging work, clinical studies are included to contextualize the broader field. Inclusion criteria: within-subject ERP–MRI correlation, cohort breadth, and methodological range (ROI, whole-brain, diffusion).

aMCI, amnestic mild cognitive impairment; AD, Alzheimer’s disease; ADHD, attention-deficit/hyperactivity disorder; ARMS, at-risk mental state; AUC, area under the curve; CSF, cerebrospinal fluid; Cz, central midline electrode; DTI, diffusion tensor imaging; ERP, event-related potential; FA, fractional anisotropy; GM, gray matter; IHTT, interhemispheric transfer time; M, mean; MCI, mild cognitive impairment; MFG, middle frontal gyrus; ns, not significant; PD, Parkinson’s disease; Pz, parietal midline electrode; ROI, region of interest; STG, superior temporal gyrus; VBM, voxel-based morphometry; WM, white matter; yr, year.

The first pattern is that structure–function coupling emerges with age. Associations are typically weak or absent in younger samples and become detectable in older cohorts, where greater inter-individual variance in cortical morphology provides the dynamic range needed to resolve the relationship (Fjell et al., 2007; Overbye et al., 2018). If both GM and WM are increasing in variance as a function of age, albeit affected by different factors and trajectories, then the existence of shared cognitive changes supports an underlying structure–function relationship (Figure 1).

**Figure 1:**
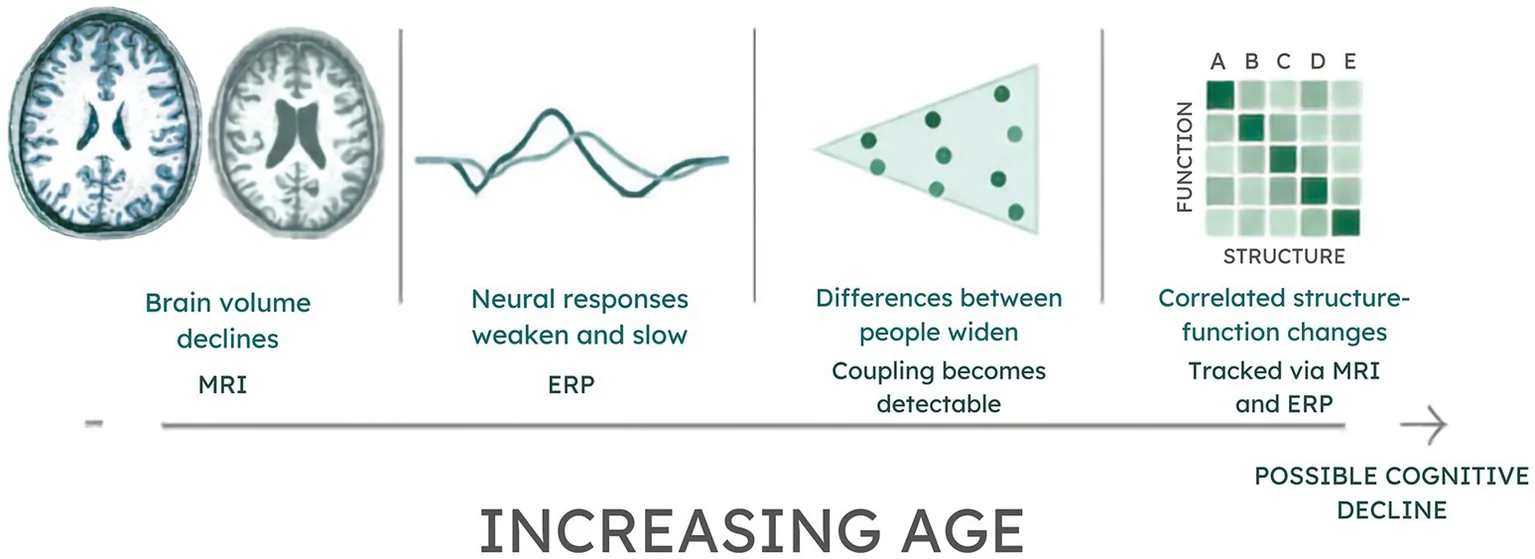
Schematic summary of age-related changes across adulthood, illustrating declining brain volume, weakening ERP responses, widening individual differences, and detectable structure–function correlations, alongside possible cognitive decline. Icons are illustrative and no values are plotted.

The second reflects a foundational principle in human electrophysiology, that an ERP response is not necessarily generated by the tissue beneath the recording electrode. Accordingly, Ford et al. (1994) found no direct spatial relationship between P300 amplitude and the GM underlying the electrode. Whole-brain analyses support this distributed pattern. For instance, P300 amplitude and latency correlated with parietal, temporal, occipital, and thalamic GM volumes (Pergher et al., 2019), while P3b latency correlated with WM across frontal, callosal, and temporal regions, alongside the thalamus and lenticular nucleus (Cardenas et al., 2005).

The third is that the brain regions changing in association with ERP responses depend also on the specific task used to elicit them. For the P300, for example, amplitude changes in automatic-attention paradigms tend to involve frontal GM, while effortful paradigms implicate parietal GM (Ford et al., 1994). The same logic extends to the sub-component level for the P300a and P300b. Fjell et al. (2007) used the P300a and P300b sensitivity to novel and target stimuli, respectively, to characterize different cortical territories in the same older cohort. These findings support a broader principle, that which anatomical change tracks a given ERP change is set by the cognitive operation engaged, not the ERP alone.

The fourth concerns a common framework in which ERP amplitude depends on GM integrity and latency on WM conduction speed. Amplitude reflects the number of pyramidal neurons synchronously activated during summation of post-synaptic potentials in GM, and therefore indexes the amount of processing activity (Luck, 2014). Latency, by contrast, indexes processing speed, which depends largely but not exclusively on WM. The joint decline of P300 amplitude and latency with age highlights the difficulty of partitioning the two (Cardenas et al., 2005; Polich, 2007; van Dinteren et al., 2014). Accordingly, reductions in GM volume have been shown to correlate with delays in P300 peak latency (Fjell et al., 2007; Pergher et al., 2019), and WM changes have similarly been linked to P300 amplitude reductions (Kemmotsu et al., 2012). In a clinical cohort, Fusar-Poli et al. (2011) found P300 amplitude tracking corpus callosum and arcuate fasciculus WM rather than GM. The scalp-recorded response depends not only on localized activation of cortical sources but also on the temporal coordination of those sources across the pathways linking them (Nunez and Srinivasan, 2006).

The anterior redistribution of the P300 described earlier illustrates that aging can be conceptualized as more than decline. Compensation accounts, including the posterior–anterior shift in aging (PASA), hemispheric asymmetry reduction in older adults (HAROLD), and the compensation-related utilization of neural circuits hypothesis (CRUNCH), propose greater frontal or bilateral engagement in older adults (Cabeza, 2002; Reuter-Lorenz and Cappell, 2008; Davis et al., 2008). Regions are recruited that were not previously engaged, so the outcome is reorganization rather than attenuation. What changes with age is therefore not only how much tissue remains, but which networks contribute to a given ERP response.

Several questions remain. First, it is unclear why a given ERP response correlates more strongly with volumetric reductions in a specific region or structure. Nonetheless, the largest changes in MRI tissue volume should correlate most highly with the largest changes in the ERP response. Second, how this gradual healthy-aging pattern relates to disease, where the same structural changes occur more sharply, is unresolved. Most direct MRI-ERP evidence comes from clinical and pre-clinical cohorts complicated by disease pathology (Egan et al., 1994; Fusar-Poli et al., 2011; Hoppstädter et al., 2013; Braverman et al., 2015; Kimiskidis et al., 2016; Gao et al., 2021). This principle, that the ERP signal tracks whichever tissue varies most, holds across both literatures, but clinical cohorts are a poor place to start because aging and disease have overlapping time courses that cannot be easily separated. A healthy-aging cohort provides the critical missing baseline, isolating the coupling under normal aging from any ongoing effects of disease. No such dataset, spanning a wide age range and predominantly healthy adults, yet exists.

A final development making this timely is technology. Portable, standardized ERP systems now capture core responses at the point of care (Ighalo et al., 2024), allowing electrophysiology to be recorded alongside routine structural MRI in a single visit, at the scale a normative model requires. Artificial intelligence now integrates these multimodal streams within one workflow, with machine learning modeling structure and function jointly, capturing distributed, nonlinear dependencies that region-by-region analyses miss, and how the coupling shifts with age. This makes it newly feasible to apply the coupling not only across a group but within a single person, flagging where an individual’s structure and function diverge before clinical signs appear.

## 5 Limitations and future directions

The central limitation is how few studies have collected MRI and ERP data in the same individuals. Samples rarely exceed *n* = 70, with MRI sub-samples as small as *n* = 16, limiting power (Pergher et al., 2019). Studies also differ in the tissue examined, often GM or WM alone rather than both, in the spatial scale at which it is measured, and in the ERP task used (Ford et al., 1994; Fjell et al., 2007; Riedel et al., 2024).

Age is handled inconsistently, and this is the most consequential gap. Studies dichotomize into young versus elderly groups (Fjell et al., 2007; Kemmotsu et al., 2012), compare narrow age bands within older adulthood (Pergher et al., 2019), examine a single age range (Hoppstädter et al., 2013; Gao et al., 2021; Riedel et al., 2024), or treat age as a control variable (Ford et al., 1994; Braverman et al., 2015). None samples continuously across a broad adult range, leaving untested whether the canonical couplings hold across adulthood and whether the coupling itself strengthens, weakens, or shifts with age. This is best answered with continuous age sampling in cross-sectional designs.

The structure–function relationships in this review are based almost entirely on the P300. Whether other components couple to structure in comparable ways is largely unknown. Resolving this would require eliciting additional components within the same acquisition, as existing protocols spanning the sensory-to-semantic hierarchy already allow (Ghosh Hajra et al., 2016).

Recent work has paired MRI and ERP measures as parallel inputs for disease classification, relating each modality to diagnostic status rather than to one another (Song et al., 2026). These models indicate whether disease is present, but not what change underlies it, since each measure is a separate predictor. Characterizing the MRI–ERP relationship itself, rather than the separate diagnostic value of each modality, requires studying it directly in healthy aging.

The principle described here is interpretive rather than predictive. It makes sense of the couplings reported across existing studies but cannot say beforehand which structure will couple to a given ERP measure in a new sample. Making it predictive would require a normative model of structure–function coupling in healthy aging, built from an ERP-MRI dataset, one that specifies, across the adult age range, which structures couple most strongly to a given ERP measure and how much variation around that coupling is normal. An individual assessed against such a model could then be placed relative to the healthy range, and a deviation from the expected coupling would mark where structure and function have begun to dissociate, the basis on which preclinical detection rests.

## 6 Conclusion

Structural and electrophysiological brain aging are each well characterized, but the relationship between them is not, and the studies that examine it directly appear to conflict. This review has argued that the conflict is apparent. The structural correlate of an ERP measure is contextual rather than fixed, and the scatter across studies is the expected signature of a context-dependent relationship read across heterogeneous designs. Establishing that relationship directly, in healthy adults across a broad age range where preclinical change first emerges, is what would allow ERP and MRI to be read as complementary measures of brain aging rather than independent ones.
